# Edentulism and Its Rehabilitation Among Older People in China

**DOI:** 10.1111/ger.70015

**Published:** 2025-09-19

**Authors:** Qiuping Zhou, Reinhard Chun Wang Chau, Hui Min Chen, Hui Chen, Colman McGrath

**Affiliations:** ^1^ Department of Dental Public Health Faculty of Dentistry, The University of Hong Kong Hong Kong SAR People's Republic of China; ^2^ Division of Restorative Dental Sciences Faculty of Dentistry, The University of Hong Kong Hong Kong SAR People's Republic of China

**Keywords:** China, edentulism, older population, rehabilitation

## Abstract

**Objectives:**

To determine the prevalence of edentulism and its rehabilitation in China's older population and describe socio‐demographic differences in edentulism and rehabilitation.

**Methods:**

Secondary analyses of the China Health and Retirement Longitudinal Study (CHARLS). Bivariate and regression analyses were performed.

**Results:**

The weighted prevalence of edentulism was 26.0% (95% CI 24.8–27.2). Approximately two‐thirds of edentulous elders had been rehabilitated (63.6%, 95% CI 57.4–69.4). In regression analyses, older people had a higher prevalence ratio (PR) for edentulism than younger elders: PR 3.0 (95% CI 2.7–3.4) for those aged 80 and older and PR 1.9 (95% CI 1.7–2.1) for those aged 70–79, with reference to those aged 60–69. Those who had attained secondary education had a lower PR for edentulism than those with no formal education: PR 0.7 (95% CI 0.6–0.8). Those from Non‐Agricultural Hukou (Urban area) had a lower PR than those from Agricultural Hukou (Rural area): PR 0.8 (95% CI 0.7, 1.0). Older adults aged 80 and above were less likely to receive denture rehabilitation compared to those aged 60–69, with a PR of 0.6 (95% CI: 0.5–0.9). In contrast, individuals with secondary education showed a greater likelihood of denture rehabilitation than those without formal education, with a PR of 1.3 (95% CI: 1.0–1.6). Additionally, non‐Han ethnic groups had a slightly higher chance of denture rehabilitation compared to their Han counterparts, with a PR of 1.2 (95% CI: 1.0–1.5).

**Conclusions:**

Approximately one in four older people in China are edentulous, and approximately two in three of them have been rehabilitated. Socio‐demographic differences in edentulism rates and rates of rehabilitation are apparent. These findings reveal disparities in edentulism and denture rehabilitation among China's older adults (60+), urging targeted policies to enhance access for less‐educated and rural elders while offering insights for global ageing populations.

## 1 Introduction

The world's population is ageing rapidly. Between 2015 and 2050, the number of people aged 60 and over is projected to increase from 900 million to 2 billion [1]. By 2050, 80% of older people are expected to live in low‐ and middle‐income countries [1]. China entered into an ‘ageing society’ in 1999—the first major developing country to do so—and now has one of the largest older populations in the world [2, 3, 4]. As of 2018, the population aged 60 and above in China reached 250 million, accounting for 17.9% of its overall population, while the population aged 65 and above reached more than 170 million, accounting for 11.9% [5]. In 2022, the proportion of people aged 65 and above in China reached 14.9%, and the ratio of the aged population was higher than the world average [6]. At present, China's population is ageing at an unprecedented rate.

With the increasing ageing of the population, the incidence of chronic diseases is increasing yearly, and this has become a critical public health issue. The complete loss of all natural teeth from the mouth is viewed as the endpoint of multifactorial oral diseases and other comorbidities. Edentulism serves as a critical indicator of cumulative oral health disease burden [7]. It can lead to impaired masticatory function (hampered chewing ability), increased loss of oral‐facial structure (accelerated facial structure changes) and deficiencies in oral mucosal protection mechanisms (weakened oral tissue protection) [8]. It is also associated with poorer overall health and quality of life [9, 10]. Therefore, a comprehensive understanding of the epidemiology of total tooth loss in China is important to inform oral health strategies for older people. Oral rehabilitation is necessary since the replacement of tooth loss by dental prostheses can help restore chewing, promote better nutrition and aesthetics and improve quality of life and well‐being [11]. As a result, there is a need to understand the current challenge, particularly variations and inequalities in edentulism and rehabilitation.

By 2012, the prevalence of edentulism was 8.64% among Chinese adults aged 45 and above, with older age being a robust predictor [12]. Thus, with the increasing number and proportion of older people in China, the health of older adults has become an important topic for ‘Healthy China’ [5]. To this end, one of the largest worldwide epidemiological studies has been carried out. The China Health and Retirement Longitudinal Study (CHARLS) is a large prospective study of Chinese community residents ≥ 45 years old [13]. The fourth wave of the survey, launched in 2018, provides for the first time data on both tooth loss and its rehabilitation. The data was released for secondary analyses in 2023 [14].

This study investigated the occurrence and sociodemographic association of self‐reported edentulism and the utilisation of removable dental prostheses for rehabilitation among individuals aged 60 and above in China.

## 2 Materials and Methods

### 2.1 Data Source and Sampling Method

The China Health and Retirement Longitudinal Study (CHARLS) is a large prospective study of Chinese community residents ≥ 45 years old, jointly conducted by the National School of Development of Peking University, the China Social Science Survey Center of Peking University, and the Youth League Committee of Peking University [13]. The CHARLS used a complex, multistage design to create a nationally representative sample of community‐dwelling Chinese adults over the age of 45 years. The survey used probability‐proportional‐to‐size sampling and stratification by region, urban/rural counties and per capita gross domestic product (GDP). Full details on the survey design have been published elsewhere [14]. In secondary analyses, the data used come from the fourth wave of the CHARLS survey data in 2018, as it contained for the first time oral health data, both on tooth loss and rehabilitation of tooth loss (data released in 2023). The focus is on those aged 60 and older, specifically the Chinese older population. Included in the content analyses were those who: (i) were aged ≥ 60 years, (ii) completed the oral health questions, and (iii) had complete socio‐demographic information.

### 2.2 Assessment of Key Outcomes

The dependent variables for this study were edentulism and rehabilitation. Edentulism was defined as having lost all of one's natural teeth, and was assessed by asking, ‘Have you lost all of your teeth?’ Participants were categorised as ‘dentate’ or ‘edentate’ according to their response of ‘no’ or ‘yes’, respectively [14]. For rehabilitation of edentulism, participants were asked, ‘Do you wear a removable dental prosthesis (i.e., denture)?’ Based on the ‘yes’ and ‘no’ answers, the participants were then divided into ‘rehabilitation’ and ‘no rehabilitation’ groups [14].

### 2.3 Assessment of Socio‐Demographic Characteristics

Age, gender, education level, economic status, residency (Hukou) and ethnicity were obtained from participants' self‐reports. Age was classified into three groups (60–69, 70–79 and 80+) based on age at the time of the 2018 survey. Educational attainment level was classified as no formal education, primary school or secondary school and above. The economic situation of participants was assessed by categorising monthly income as lower income (≤ 1000 RMB) or higher income (> 1000 RMB) (The monthly income consists of the sum of seven parts. Part 1: Pension for Public Servants, Public Institution Employees, and Basic Pension for Enterprise Employees. Part 2: Supplementary Pension Insurance (Annuity). Part 3: Urban and Rural Resident Pension, New Rural Resident Pension and Urban Resident Pension. Part 4: Pension for Land‐Expropriated Farmers. Part 5: Life Insurance. Part 6: Commercial Pension Insurance. Part 7: Other Pension. For any individual, monthly income may be one or more of these.). Residency (Hukou), China's household registration system, which classifies individuals as agricultural (rural) or non‐agricultural (urban) based on their registered place of residence, impacts access to services like healthcare. In the CHARLS survey, it was classified as ‘agricultural’ (rural) or ‘non‐agricultural’ (urban) based on participants' household registration status by the time of questionnaire completion. Ethnicity was classified as Han Chinese or non‐Han Chinese.

### 2.4 Data Analyses

The data were analysed using IBM SPSS Statistics for Windows, version 28.0 (IBM Corp., Armonk, NY, USA). The data were weighted with corrections for household and individual non‐response (INDV_weight_ad2). Frequency tables were produced of weighted percentages and 95% confidence intervals (CI) by socio‐demographic characteristics of the sample. Bivariate analyses of differences in edentulism by age, gender, education level, economic status, Hukou and ethnicity were examined through cross‐tabulations in SPSS. Similarly, differences in rehabilitation with respect to age, gender, education level, economic status, Hukou and ethnicity were examined through cross‐tabulation in SPSS. Two Poisson regression analyses were then performed. In the first model, factors associated with edentulism were determined. The dependent variable was edentulism (0 = no, 1 = yes) and the independent variables were age group (60–69, 70–79, > 80), gender (male, female), educational attainment level (No formal education, Primary, Secondary or above), monthly income (≤ 1000 RMB, > 1000 RMB), Hukou (agricultural, non‐agricultural) and ethnicity (Han, non‐Han). In the second model, factors associated with rehabilitation of edentulism were determined in Poisson regression analyses. The dependent variable was rehabilitation (0 = no, 1 = yes) and the independent variables were age group (60–69, 70–79, > 80), gender (male, female), educational attainment level (No formal education, Primary, Secondary or above), monthly income (≤ 1000 RMB, > 1000 RMB), Hukou (agricultural, non‐agricultural) and ethnicity (Han, non‐Han).

## 3 Results

**FIGURE 1:**
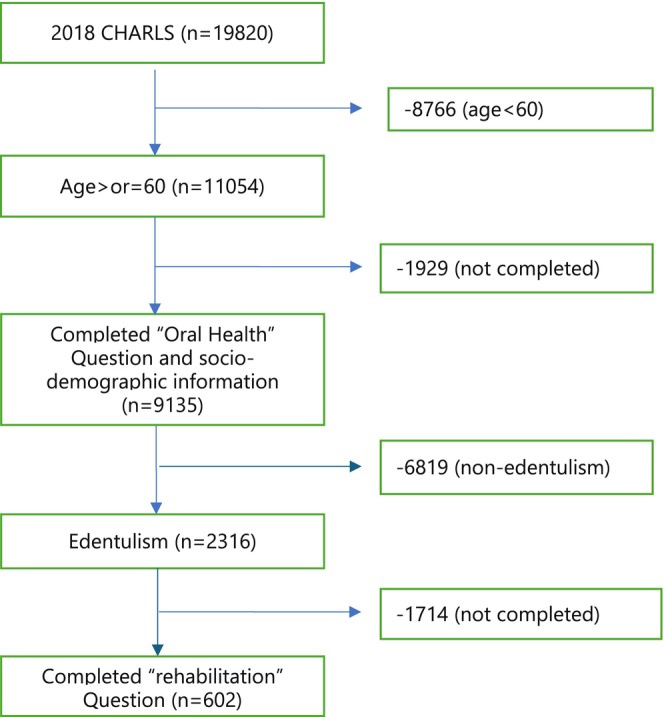
Flowchart of data selection for analyses. [Colour figure can be viewed at wileyonlinelibrary.com]

The 4th wave of CHARLS, conducted in 2018, had 19,820 participants. Of these, 55.8% (11,054) were aged 60 years or older. Among them, 82.6% (9135/11,054) provided information on their dentate status (‘Have you lost all of your teeth?’ Yes/No) and provided information on socio‐demographic characteristics. Among those (*n* = 2316) who reported being edentulous, 602 provided information on whether they had a denture or not (Do you wear a removable dental prosthesis (i.e., denture)? Yes/No). A flowchart illustrating the selection of records identified, included and excluded, with reasons, is presented in Figure 1.

The weighted prevalence of edentulism was 26.0% (95% CI 24.8–27.2). The socio‐demographic profile of participants (weighted percentage) is presented in Table 1. Over half the weighted sample were aged 60–69 (55.4%, CI 54.1, 56.8). Approximately half of the participants were female (50.1%, 95% CI 48.7, 51.5). Approximately half the weighted proportion reported receiving no formal education (48.7%, 95% CI 47.3, 50.0). The weighted proportion of Han participants was 93.7% (95% CI 93.0, 94.3). The weighted proportion of those who reported monthly incomes equal to or below 1000 RMB was 65.3% (95% CI 63.8, 66.7). The weighted percentage of those reporting to have an agricultural Hukou was 66.4% (95% CI 65.0, 67.9). The weighted percentage profile of the edentulous sample who completed the rehabilitation questionnaire was similar to the weighted percentage profile of the edentulous sample overall (Table 1).

**TABLE 1:** Profiles of participants.

Variations	Included sample (*n* = 9135)	Edentulism and completed rehabilitation question (*n* = 602)
	Weighted % (95% CI)	Weighted % (95% CI)
Age		
60–69	55.4 (54.1–56.8)	46.8 (40.2–53.5)
70–79	30.8 (29.6–32.1)	36.4 (30.9–42.3)
80+	13.8 (12.8–14.8)	16.8 (12.9–21.6)
Gender		
Male	49.9 (48.5–51.3)	48.2 (41.7–54.9)
Female	50.1 (48.7–51.5)	51.8 (45.1–58.3)
Education		
No formal education	48.7 (47.3–50.0)	58.4 (51.2–65.2)
Primary	21.4 (20.3–22.5)	21.0 (16.2–26.6)
Secondary or above	30.0 (28.6–31.4)	20.7 (14.0–29.4)
Income		
Lower (≤ 1000)	65.3 (63.8–66.7)	69.3 (61.0–76.6)
Higher (> 1000)	34.7 (33.3–36.2)	30.7 (23.4–39.0)
Hukou		
Agricultural	66.4 (65.0–67.9)	70.7 (62.2–78.0)
Non‐agricultural	33.6 (32.1–35.0)	29.3 (22.0–37.8)
Ethnicity		
Han	93.7 (93.0–94.3)	93.7 (90.3–96.0)
Non‐han	6.3 (5.7–7.0)	6.3 (4.0–9.7)

Socio‐demographic differences in edentulism were apparent (Table 2). Reported edentulism varied significantly by age, education, income and Hukou status (*p* < 0.001). Prevalence increased markedly with age, from 16.6% in those aged 60–69 to 52.3% in those 80 and older. Women reported higher rates than men (27.9% vs. 24.1%). Education level showed a clear gradient: 32.2% of those with no formal education were edentulous, compared to 25.6% with primary school education and 16.2% with secondary school or higher. Income also played a role, with 29.7% of the lower‐income group affected versus 19.1% of the higher‐income group. Edentulism was more common among those with agricultural Hukou (29.5%) than non‐agricultural Hukou (19.1%). No significant difference was found between Han and non‐Han ethnic groups (26.0% vs. 26.7%, *p* > 0.05).

**TABLE 2:** Socio‐demographic variations in tooth loss (weighted estimates).

Variations	Edentulism weighted % (95% CI)	Non‐edentulism Weighted % (95% CI)
Age		
60–69	16.6 (15.3–18.1)	83.4 (81.9–84.8)
70–79	31.2 (29.1–33.3)	68.9 (66.7–70.9)
> 80	52.3 (48.3–56.3)	47.7 (43.7–51.7)
Gender		
Male	24.1 (22.4–25.9)	75.9 (74.2–77.7)
Female	27.9 (26.3–29.6)	72.1 (70.4–73.7)
Education		
No formal education	32.2 (30.6–33.8)	67.8 (66.2–69.4)
Primary	25.6 (23.1–28.3)	74.4 (71.7–76.9)
Secondary or above	16.2 (14.0–18.7)	83.8 (81.3–86.0)
Income		
Lower (≤ 1000)	29.7 (28.4–31.0)	70.3 (69.0–71.6)
Higher (> 1000)	19.1 (16.9–21.7)	80.9 (78.4–83.2)
Hukou		
Agricultural	29.5 (28.2–30.8)	70.5 (69.2–71.8)
Non‐agricultural	19.1 (16.7–21.7)	80.9 (78.3–83.3)
Ethnicity		
Han	26.0 (24.7–27.2)	74.1 (72.8–75.3)
Non‐han	26.7 (22.5–31.5)	73.3 (68.5–77.6)

In Poisson regression analyses, the key factors associated with reported edentulism were age group, educational attainment and Hukou group (Table 3). Significant differences in edentulism prevalence were observed by age, education and Hukou status. Compared to those aged 60–69, individuals aged 70–79 had a 1.9‐fold higher prevalence (95% CI 1.7–2.1, *p* < 0.001), and those 80 or older had a 3.0‐fold higher prevalence (95% CI 2.7–3.4, *p* < 0.001). Those with secondary school education or higher had a 30% lower prevalence than those with no formal education (PR = 0.7, 95% CI 0.6–0.8, *p* < 0.001). Non‐agricultural Hukou individuals showed a 20% lower prevalence than those with agricultural Hukou (PR = 0.8, 95% CI 0.7–1.0, *p* < 0.05). No significant differences were found by gender, income or ethnicity.

**TABLE 3:** Socio‐demographic variations in tooth loss: Robust Poisson regression.

Variations	PR (95% CI for PR)	*p*
Age		**< 0.001**
60–69^a^	1.00	
70–79	1.86 (1.67–2.08)	**< 0.001**
> 80	3.02 (2.69–3.40)	**< 0.001**
Gender		
Male^a^	1.00	
Female	1.06 (0.97–1.16)	0.175
Education		**< 0.001**
No formal education^a^	1.00	
Primary	0.92 (0.82–1.03)	0.141
Secondary or above	0.72 (0.62–0.83)	**< 0.001**
Income		
Lower (≤ 1000)^a^	1.00	
Higher (> 1000)	0.86 (0.73–1.01)	0.068
Hukou		
Agricultural^a^	1.00	
Non‐agricultural	0.81 (0.68–0.96)	**0.017**
Ethnicity		
Han^a^	1.00	
Non‐han	1.06 (0.90–1.25)	0.466

*Note:* Bold values (*p* < 005).

Abbreviation: PR, prevalence ratio.

^a^ Reference group.

Socio‐demographic differences in rehabilitation of edentulism were apparent (Table 4). There were differences in reported rehabilitation of edentulism by age group. The weighted proportion of reported tooth loss rehabilitation was 72.5% among those aged 60–69, 61.2% among those aged 70–79, and 44.0% among those aged 80 and older. The weighted proportion of reported rehabilitation was lowest among those who claimed they had no formal education and highest among those who had obtained a secondary school level of education or above. There were similar differences by income group. Whereas the weighted proportion of reported rehabilitation of edentulism was higher among those with monthly incomes > 1000 RMB than among those with less. There were also differences by ethnicity, with it being higher among non‐Han Chinese. There were no significant differences by gender or Hukou residency.

**TABLE 4:** Socio‐demographic variations in rehabilitation of edentulism: Bivariate Analyses (weighted estimates).

Variations	No rehabilitation weighted % (95% CI)	Rehabilitation weighted % (95% CI)
Age		
60–69	27.5 (19.0–38.0)	72.5 (62.0–81.0)
70–79	38.8 (31.2–46.9)	61.2 (53.1–68.8)
> 80	56.0 (42.4–68.7)	44.0 (31.3–57.6)
Gender		
Male	35.3 (27.0–44.5)	64.7 (55.5–73.0)
Female	37.4 (29.8–45.7)	62.6 (54.3–70.2)
Education		
No formal education	42.2 (36.2–48.3)	57.9 (51.7–63.8)
Primary	35.1 (21.3–52.0)	64.9 (48.1–78.8)
Secondary or above	21.4 (12.0–35.2)	78.6 (64.8–88.0)
Income		
Lower (≤ 1000)	38.9 (33.9–44.3)	61.0 (55.7–66.1)
Higher (> 1000)	30.6 (17.8–47.3)	69.4 (52.7–82.2)
Hukou		
Agricultural	38.2 (33.2–43.4)	61.8 (56.6–66.8)
Non‐agricultural	32.0 (18.5–49.5)	68.0 (50.5–81.6)
Ethnicity		
Han	37.6 (31.5–44.1)	62.4 (55.9–68.5)
Non‐han	18.4 (7.3–39.1)	81.6 (60.9–92.7)

In Poisson regression analyses, the key factors associated with reported rehabilitation of edentulism were age group, education and ethnicity (Table 5). Those aged 80 and older had a 40% lower prevalence ratio of being rehabilitated than those aged 60–69 (PR = 0.6, 95% CI 0.5, 0.9). Those who reported having received secondary education or above had approximately a third higher prevalence ratio of being rehabilitated than those reporting having no formal education (PR = 1.3, 95% CI 1.0, 1.6). Those of non‐Han ethnicity had a higher PR than those of Han ethnicity (PR 1.2, 95% CI 1.0, 1.5). There was no significant difference in the rehabilitation of edentulism with respect to gender, income or Hukou type.

**TABLE 5:** Socio‐demographic variations in rehabilitation of edentulism: Robust Poisson regression.

Variations	PR (95% CI for PR)	*p*
Age		**0.011**
60–69^a^	1.00	
70–79	0.84 (0.70–1.01)	0.057
> 80	0.64 (0.47–0.88)	**0.006**
Gender		
Male^a^	1.00	
Female	1.02 (0.85–1.24)	0.819
Education		0.101
No Formal Education^a^	1.00	
Primary	1.09 (0.84–1.41)	0.505
Secondary or above	1.27 (1.01–1.60)	**0.040**
Income		
Lower (≤ 1000)^a^	1.00	
Higher (> 1000)	1.16 (0.88–1.54)	0.287
Hukou		
Agricultural^a^	1.00	
Non‐agricultural	0.86 (0.65–1.14)	0.296
Ethnicity		
Han^a^	1.00	
Non‐han	1.23 (1.01–1.49)	**0.036**

*Note:* Bold values (*p* < 005).

Abbreviation: PR, prevalence ratio.

^a^ Reference group.

## 4 Discussion

The 4th wave of the CHARLS study revealed that approximately 25% of individuals aged 60 years and older in China were edentulous, based on self‐reported data. This prevalence is higher than many global estimates but lower than earlier surveys in China, suggesting improvements in oral healthcare services. Multivariate analyses identified significant associations between edentulism and age, educational attainment and Hukou residency status. About two‐thirds of edentulous individuals used dentures for rehabilitation, with variations by age, education and ethnicity.

The CHARLS dataset is a robust resource for studying ageing in China, offering comprehensive health and socioeconomic data. Its large sample size and weighted adjustments for non‐response enhance representativeness. However, limitations include the limited availability of oral health‐specific assessments, potential response bias due to incomplete responses, and the cross‐sectional design, which precludes causal inferences. Additionally, the data, collected in 2018 and released in 2023, may not accurately reflect recent disruptions, such as those caused by COVID‐19, that have affected dental service access.

Compared to global studies, the higher edentulism prevalence in China aligns with findings from countries with limited dental care access [15, 16, 17]. The observed decline in edentulism compared to prior Chinese surveys [12] suggests progress in oral healthcare infrastructure, contrasting with earlier reports of higher tooth loss. The association between edentulism and age is consistent with global trends [18], but the influence of education and Hukou status highlights unique socioeconomic factors in China [19, 20, 21, 22, 23, 24]. Discrepancies in denture use compared to institutionalised populations underscore setting‐specific differences, with China's community‐dwelling older adults showing higher rehabilitation rates [25, 26].

The higher edentulism prevalence in China likely stems from disparities in dental care access, influenced by socioeconomic factors such as education and Hukou status. Lower educational attainment correlates with reduced oral health awareness and care‐seeking behaviour, while rural (agricultural Hukou) residents face barriers in accessing quality dental services [22, 23, 24, 27, 28]. The higher denture use among educated and non‐Han Chinese individuals suggests socioeconomic and cultural influences on rehabilitation uptake [29, 30]. For clinicians and policymakers, these findings highlight the need to address inequities in oral healthcare access, particularly for older, less educated and rural populations. Emerging technologies, such as AI‐powered mHealth platforms, could facilitate remote screening and improve access for underserved groups [31, 32, 33].

The study raises questions about the current state of edentulism and rehabilitation post‐COVID‐19, given the 2018 data collection. Longitudinal studies are needed to establish causality and track changes in oral health outcomes. Further research should explore specific barriers to dental care access in rural areas and among less‐educated groups, as well as the effectiveness of mHealth interventions in reducing disparities. Additionally, more detailed oral health assessments in future CHARLS waves could enhance the understanding of these trends.

## 5 Conclusion

Approximately one in four older people in China report having lost all their teeth. Among those reporting to be edentulous, approximately two‐thirds claim to be rehabilitated by dentures. Socio‐demographic variations exist concerning tooth loss and rehabilitation. Age, education level and residency (Hukou) are key factors associated with total tooth loss. Age, education level and ethnicity are key factors associated with the rehabilitation of tooth loss. These findings have implications for oral health policy and practice for older people in China and beyond.

## Author Contributions

**Qiuping Zhou:** conceptualization, data curation, formal analysis, methodology, visualization, writing – original draft, writing – review and editing. **Reinhard Chun Wang Chau:** data curation, formal analysis, methodology, validation, writing – original draft, writing – review and editing. **Hui Min Chen:** data curation, formal analysis, writing – review and editing. **Hui Chen:** supervision, writing – review and editing. **Colman McGrath:** conceptualization, methodology, project administration, supervision, validation, writing – review and editing.

## Ethics Statement

This study utilised the China Health and Retirement Longitudinal Study (CHARLS) dataset, which is publicly available and fully anonymised. As this study involves secondary analysis of de‐identified data, no additional ethics approval was required.

## Conflicts of Interest

The authors declare no conflicts of interest.

## Data Availability

The data used in this study are from the China Health and Retirement Longitudinal Study (CHARLS), publicly available at http://charls.pku.edu.cn. The dataset is accessible to researchers upon registration with the National School of Development, Peking University, under a Creative Commons Attribution 4.0 International (CC BY 4.0) licence.

## References

[1] J. R. Beard , A. Officer , I. A. de Carvalho , et al., “The World Report on Ageing and Health: A Policy Framework for Healthy Ageing,” Lancet 387, no. 10033 (2016): 2145–2154, 10.1016/s0140-6736(15)00516-4. 26520231 PMC4848186

[2] L. Wu , Z. Huang , and Z. Pan , “The Spatiality and Driving Forces of Population Ageing in China,” PLoS One 16, no. 1 (2021): e0243559, 10.1371/journal.pone.0243559. 33428682 PMC7799793

[3] M. F. Krings , J. D. H. van Wijngaarden , S. Yuan , and R. Huijsman , “China's Elder Care Policies 1994–2020: A Narrative Document Analysis,” International Journal of Environmental Research and Public Health 19, no. 10 (2022): 6141, 10.3390/ijerph19106141. 35627677 PMC9141963

[4] Z. Feng , E. Glinskaya , H. Chen , et al., “Long‐Term Care System for Older Adults in China: Policy Landscape, Challenges, and Future Prospects,” Lancet 396, no. 10259 (2020): 1362–1372, 10.1016/s0140-6736(20)32136-x. 34338215

[5] X. Chen , J. Giles , Y. Yao , et al., “The Path to Healthy Ageing in China: A Peking University‐Lancet Commission,” Lancet 400, no. 10367 (2022): 1967–2006, 10.1016/s0140-6736(22)01546-x. 36423650 PMC9801271

[6] Z. Feng , J. Falkingham , X. Liu , and A. Vlachantoni , “Changes in Living Arrangements and Mortality Among Older People in China,” SSM—Population Health 3 (2017): 9–19, 10.1016/j.ssmph.2016.11.009. 29349200 PMC5768996

[7] Global Burden Oral Disorders Collaborators , E. Bernabe , W. Marcenes , et al., “Global, Regional, and National Levels and Trends in Burden of Oral Conditions From 1990 to 2017: A Systematic Analysis for the Global Burden of Disease 2017 Study,” Journal of Dental Research 99 (2020): 362–373, 10.1177/0022034520908533. 32122215 PMC7088322

[8] Y. Fan , X. Shu , K. C. M. Leung , and E. C. M. Lo , “Association Between Masticatory Performance and Oral Conditions in Adults: A Systematic Review and Meta‐Analysis,” Journal of Dentistry 129 (2023): 104395, 10.1016/j.jdent.2022.104395. 36563840

[9] D. S. Brennan , A. J. Spencer , and K. F. Roberts‐Thomson , “Tooth Loss, Chewing Ability and Quality of Life,” Quality of Life Research 17, no. 2 (2008): 227–235, 10.1007/s11136-007-9293-2. 18075784

[10] S. Gennai , R. Izzetti , M. C. Pioli , L. Music , and F. Graziani , “Impact of Rehabilitation Versus Edentulism on Systemic Health and Quality of Life in Patients Affected by Periodontitis: A Systematic Review and Meta‐Analysis,” Journal of Clinical Periodontology 49, no. Suppl 24 (2022): 328–358, 10.1111/jcpe.13526. 34761419

[11] Z. Ali , S. R. Baker , S. Shahrbaf , N. Martin , and M. V. Vettore , “Oral Health‐Related Quality of Life After Prosthodontic Treatment for Patients With Partial Edentulism: A Systematic Review and Meta‐Analysis,” Journal of Prosthetic Dentistry 121, no. 1 (2019): 59–68.e3, 10.1016/j.prosdent.2018.03.003. 30006220

[12] C. Ren , C. McGrath , and Y. Yang , “Edentulism and Associated Factors Among Community‐Dwelling Middle‐Aged and Elderly Adults in China,” Gerodontology 34, no. 2 (2017): 195–207, 10.1111/ger.12249. 27709661

[13] Y. Zhao , Y. Hu , J. P. Smith , J. Strauss , and G. Yang , “Cohort Profile: The China Health and Retirement Longitudinal Study (CHARLS),” International Journal of Epidemiology 43, no. 1 (2014): 61–68, 10.1093/ije/dys203. 23243115 PMC3937970

[14] National School of Development, Peking University , “China Health and Retirement Longitudinal Study Wave 4 User's Guide,” National School of Development, Peking University (2020), https://charls.pku.edu.cn/en/data/User2018.pdf.

[15] D. A. White , G. Tsakos , N. B. Pitts , et al., “Adult Dental Health Survey 2009: Common Oral Health Conditions and Their Impact on the Population,” British Dental Journal 213, no. 11 (2012): 567–572, 10.1038/sj.bdj.2012.1088. 23222333

[16] W. Marcenes , N. J. Kassebaum , E. Bernabé , et al., “Global Burden of Oral Conditions in 1990–2010: A Systematic Analysis,” Journal of Dental Research 92, no. 7 (2013): 592–597, 10.1177/0022034513490168. 23720570 PMC4484374

[17] S. Tyrovolas , A. Koyanagi , D. B. Panagiotakos , et al., “Population Prevalence of Edentulism and Its Association With Depression and Self‐Rated Health,” Scientific Reports 6 (2016): 37083, 10.1038/srep37083. 27853193 PMC5112530

[18] X. Qin , L. Chen , X. Yuan , et al., “Projecting Trends in the Disease Burden of Adult Edentulism in China Between 2020 and 2030: A Systematic Study Based on the Global Burden of Disease,” Frontiers in Public Health 12 (2024): 1367138, 10.3389/fpubh.2024.1367138. 38638472 PMC11024259

[19] Z. Tang , C. Huang , Y. Li , Y. Sun , and X. Chen , “Early‐Life Adversity and Edentulism Among Chinese Older Adults,” BMC Oral Health 22, no. 1 (2022): 542, 10.1186/s12903-022-02595-3. 36434640 PMC9700936

[20] X. Zhang , S. Dai , X. Jiang , W. Huang , Q. Zhou , and S. Wang , “The Pathways From Disadvantaged Socioeconomic Status in Childhood to Edentulism in Mid‐to‐Late Adulthood Over the Life‐Course,” International Journal for Equity in Health 22, no. 1 (2023): 150, 10.1186/s12939-023-01865-y. 37553562 PMC10408210

[21] L. Jiang , J. Li , Z. Yang , et al., “Analysis of Epidemiological Trends of and Associated Factors for Tooth Loss Among 35‐ to 44‐Year‐Old Adults in Guangdong, Southern China, 1995–2015: A Population‐Based Cross‐Sectional Survey,” BMC Oral Health 23, no. 1 (2023): 74, 10.1186/s12903-023-02776-8. 36740667 PMC9899388

[22] Q. Song and J. P. Smith , “Hukou System, Mechanisms, and Health Stratification Across the Life Course in Rural and Urban China,” Health & Place 58 (2019): 102150, 10.1016/j.healthplace.2019.102150. 31212169 PMC6708454

[23] H. Liu , J. A. Rizzo , and H. Fang , “Urban‐Rural Disparities in Child Nutrition‐Related Health Outcomes in China: The Role of Hukou Policy,” BMC Public Health 15 (2015): 1159, 10.1186/s12889-015-2517-4. 26596931 PMC4657335

[24] C. Li and N. A. Yao , “Socio‐Economic Disparities in Dental Health and Dental Care Utilisation Among Older Chinese,” International Dental Journal 71, no. 1 (2021): 67–75, 10.1111/idj.12600. 33616055 PMC9275339

[25] Z. Xin‐yue and L. We , “Investigation and Analysis of the Dentition, Denture Restoration and Oral Health Behavior of the Elderly Over 80 Years Old in Two Nursing Homes in Chengdu,” Beijing Journal of Stomatology 30, no. 2 (2022): 128–131.

[26] J. Guo , J. H. Ban , G. Li , et al., “Status of Tooth Loss and Denture Restoration in Chinese Adult Population: Findings From the 4th National Oral Health Survey,” Chinese Journal of Dental Research 21, no. 4 (2018): 249–257, 10.3290/j.cjdr.a41083. 30264041

[27] R. Mõttus , J. M. Starr , and I. J. Deary , “Predicting Tooth Loss in Older Age: Interplay Between Personality and Socioeconomic Status,” Health Psychology 32, no. 2 (2013): 223–226, 10.1037/a0027357. 22329425

[28] R. C. Ferreira , J. G. S. Souza , A. Soares , A. R. d. S. Soares , R. V. Vieira , and I. Kawachi , “Income‐ and Education‐Based Inequalities of Edentulism and Dental Services Utilization in Brazil,” Community Dentistry and Oral Epidemiology 51, no. 5 (2023): 829–837, 10.1111/cdoe.12771. 35801281

[29] M. Xu , M. Cheng , X. Gao , et al., “Factors Associated With Oral Health Service Utilization Among Adults and Older Adults in China, 2015–2016,” Community Dentistry and Oral Epidemiology 48, no. 1 (2020): 32–41, 10.1111/cdoe.12497. 31621099

[30] S. C. Wu , X. X. Ma , Z. Y. Zhang , et al., “Ethnic Disparities in Dental Caries Among Adolescents in China,” Journal of Dental Research 100, no. 5 (2021): 496–506, 10.1177/0022034520976541. 33283631

[31] R. C. W. Chau , K. M. Thu , A. Chaurasia , R. T. C. Hsung , and W. Y. H. Lam , “A Systematic Review of the Use of mHealth in Oral Health Education Among Older Adults,” Dental Journal 11, no. 8 (2023): 189. 10.3390/dj11080189 PMC10452984 37623285

[32] R. C. W. Chau , K. M. Thu , R. T. C. Hsung , et al., “Self‐Monitoring of Oral Health Using Smartphone Selfie Powered by Artificial Intelligence: Implications for Preventive Dentistry,” Oral Health & Preventive Dentistry 22 (2024): 5758200. 39308412 10.3290/j.ohpd.5758200 PMC11619897

[33] R. C. W. Chau , A. C. C. Cheng , K. Mao , et al., “External Validation of an AI mHealth Tool for Gingivitis Detection Among Older Adults at Daycare Centers: A Pilot Study,” International Dental Journal 75 (2025): 1970–1978. 39864975 10.1016/j.identj.2025.01.008 PMC12142741

